# A Topography Analysis Incorporated Optimization Method for the Selection and Placement of Best Management Practices

**DOI:** 10.1371/journal.pone.0054520

**Published:** 2013-01-21

**Authors:** Zhenyao Shen, Lei Chen, Liang Xu

**Affiliations:** State Key Laboratory of Water Environment Simulation, School of Environment, Beijing Normal University, Beijing, China; University of Catania, Italy

## Abstract

Best Management Practices (BMPs) are one of the most effective methods to control nonpoint source (NPS) pollution at a watershed scale. In this paper, the use of a topography analysis incorporated optimization method (TAIOM) was proposed, which integrates topography analysis with cost-effective optimization. The surface status, slope and the type of land use were evaluated as inputs for the optimization engine. A genetic algorithm program was coded to obtain the final optimization. The TAIOM was validated in conjunction with the Soil and Water Assessment Tool (SWAT) in the Yulin watershed in Southwestern China. The results showed that the TAIOM was more cost-effective than traditional optimization methods. The distribution of selected BMPs throughout landscapes comprising relatively flat plains and gentle slopes, suggests the need for a more operationally effective scheme, such as the TAIOM, to determine the practicability of BMPs before widespread adoption. The TAIOM developed in this study can easily be extended to other watersheds to help decision makers control NPS pollution.

## Introduction

Non-point source (NPS) pollution has been identified as the primary mechanism underlying water deterioration and biological diversity loss [1], [2]. Nowadays, the implementation of Best Management Practices (BMPs) is considered as an effective approach to control NPS pollution from agricultural, pasture, forest, mining and other sources [3], [4]. Generally, BMPs are divided into non-structural practices, in terms of tillage operation and nutrient management, and structural practices, such as filter strips, parallel terraces and grassed waterways [5]. Previous studies have illustrated the effects of non-structural BMPs on water quality, as described in the Clean Water Act and other laws [6]–[8]. However, the design of structural BMPs is more challenging, particularly at the regional or watershed scale.

One of the key challenges in developing an effective BMPs program is to achieve a maximum reduction in NPS loads at a minimal cost [7] and the target for improving cost-effectiveness is the systematic optimization of real-world efforts [9]. Arabi et al. [10] have showed that selection and placement of BMPs by optimization was found to be nearly 3 times more cost-effective than targeting methods for the same level of protection specified pollutants. A wide range of models and decision support systems are available for understanding the flow and pollutant transport for structural BMPs [11]. Srivastava et al. [12] coupled the Annualized Agricultural Non-Point Source model and a genetic algorithm for the assignment of BMPs in the field. Bekele and Nicklow [13] combined the Soil and Water Assessment Tool (SWAT) with a multi-objective evolutionary algorithm to provide tradeoffs between agricultural production and ecosystem service. Recently, the type and location of structural BMPs are two key decision variables that have been considered in watershed programs [14]. Cools et al. [15] applied a cost-effective modeling approach to an in-stream Total Maximum Daily Loads program to reduce phosphorus (P) and nitrogen (N) loads. Kaini et al. [5] designed the type, size, and location for several structural BMPs at a watershed scale. Recent developments in mathematics and computer science have also provided new techniques for optimization designs. Several options, such as linear programming [16], Monte Carlo simulation [17], scatter search [18], Tabu search [19] and non-dominated sorted genetic algorithms [20], have been addressed to develop a cost-effectiveness strategy.

Currently, the effects of structural BMPs on water quality have been reported at both plot and field scales [21], [22]. However, over the past decade, water quality has shown little improvement at the watershed level, even after the extensive implementation of structural BMPs [6], [23]. The lack of improvement could potentially reflect improper design, insufficient investigation, poor local characteristics, uncooperative landowner and changing weather [24]. In the simplest terms, the optimization design should be the systematic optimization of real-world efforts and accommodate the desires of both the governor and the engineer (as an agency or company). Therefore, it is necessary to determine the practicability of BMPs before widespread adoption. All of the related factors, including topography, land use, and accordingly the characters of slope are static parameters that affect the site characteristics with relevance to construction [25]–[26]. Engineers consider these factors as functions of physical site characteristics, which can be evaluated through detailed site investigations or geographic information systems (GIS) [25]–[27]. In fact, topography, as captured by a digital elevation model (DEM), has already been used to capture gravitational gradients and the tendency for elevation variation at each site [26]–[27]. The GIS-based topography analyses are valuable compared with inherently subjective processes, which are susceptible to personal experience and judgment for construction conditions, particularly when structural BMPs are designed at the planning stage [28]. Therefore, engineers have recently argued to add new criteria, such as land use and topography analysis, to the cost-effectiveness goal. This highlighted a dire need for developing a topography analysis incorporated optimization method (TAIOM), particularly for complex watersheds.

The Three Gorges Project, situated at Sandoupin in China, is the largest hydropower project in the world. The presence of NPS pollution due to the massive use of fertilizers at the Three Gorges Reservoir Region has been of concern to the public in recent years [29]. However, the optimization design of BMPs is relatively poorly documented in such an important watershed. The objective of this paper is to contribute information to ongoing work on the optimization method for the selection and placement of BMPs. A key aim is to develop a TAIOM to provide economic, environmental and operational effectiveness related to the optimization method. To this end, the following tasks were performed: 1) the baseline loads and spatial distributions of sediment, N and P were qualified using SWAT; 2) an allele set containing the removal efficiency and cost for each BMP combination was developed; 3) a GIS-based topography analysis was incorporated; and 4) these mentioned components were integrated using a genetic algorithm program in the Yulin watershed, China. The description of study area and related method could be found in the next section.

## Materials and Methods

### Study Watershed

**Figure 1:**
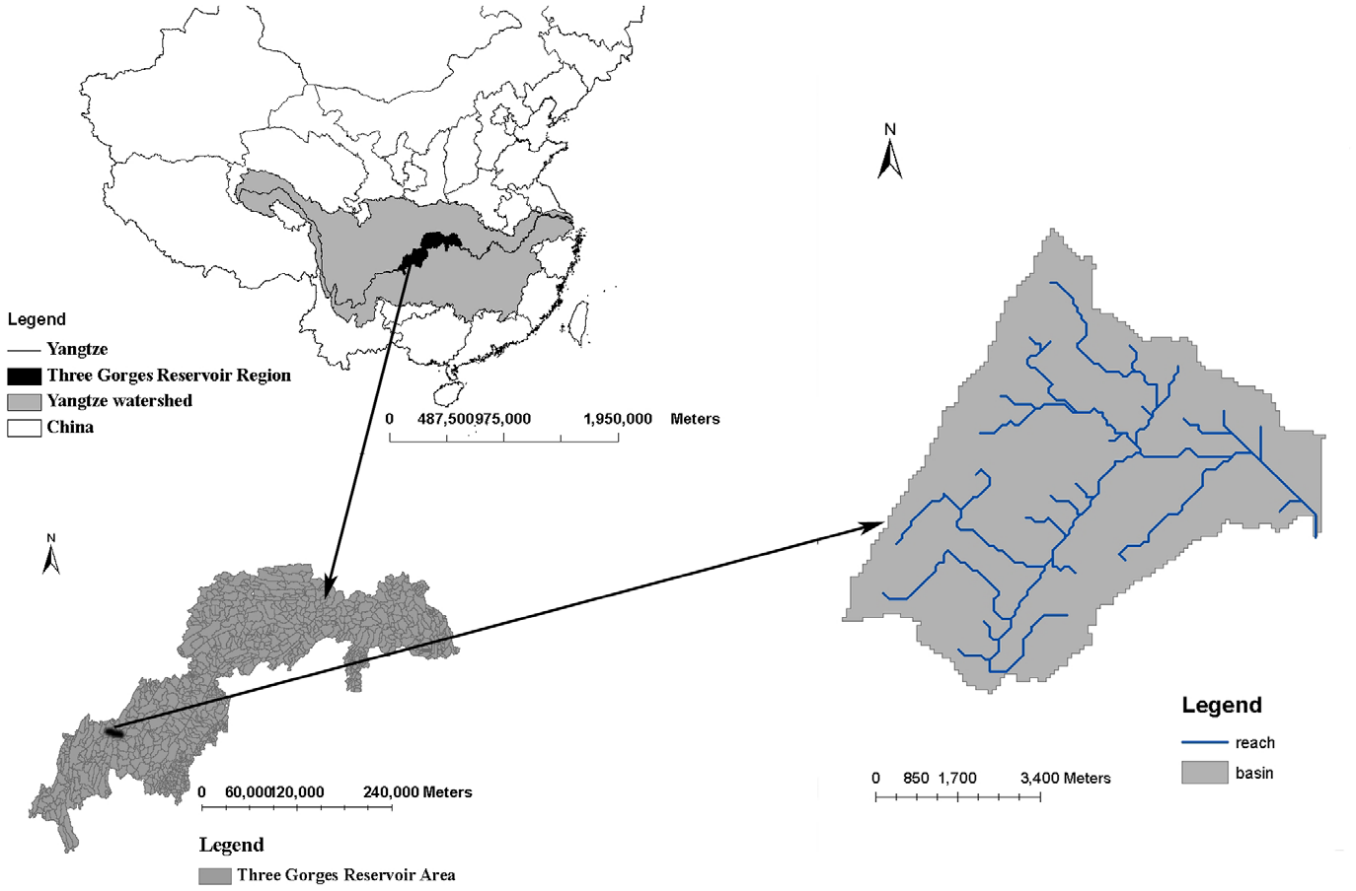
The location of the Yulin River watershed.

A sub-watershed of the Yulin River was selected as the study area (Fig. 1). The Yulin River is located in the northeast region of the district of Chongqing municipality, China. The river originates from the GaoZhai Mountains with an averaged elevation of 845 m, travels a basin that includes mountain terrain and agricultural plains, and eventually influxes to the Yangtze River, which is one of the most significant ecosystems worldwide [29]. The water quality in this river primarily reflects the construction of the Three Gorges Reservoir. Over the past 10 years, a large discharge of N and P from the Yulin watershed has caused on-site environment degradation and off-site problems associated with the downstream eutrophication of the Three Gorges Reservoir [29]. The watershed used in this study, with a drainage area of 47.24 km^2^, is a mixed land use area, of which 68.66% is covered by agricultural land (paddy and drylands), 22.38% is forest and 8.94% is bare land. Purple (15.6%), paddy (30.2%), yellow brown (26.5%) soil types were used in this study. The subtropical climate features of this watershed include an annual temperature ranging from 6.3–27.5°C and precipitation of approximately 1145.86 mm.

The details of the watershed features and the authority who issued the permission for information are listed in Table 1. We obtained the major GIS input files and the related physical data from Institute of Geographical and Natural Resources Research, Institute of Soil Science and China Meteorological Administration. The periodic monitoring flow and water quality data were obtained from local government. All necessary permits were obtained for the input data.

**Table 1:** The type and sources of available data in the Yulin watershed.

Data type	Scale	Resolution	Data description	Source
Digital Elevation Model	1∶250,000	90×90 m	Elevation, overland and channel slopes and lengths	Institute of Geographical and Natural Resources Research, Chinese Academy of Sciences; National Geomatics Center of China
Land use	1∶100,000	30×30 m	Land use classifications	Institute of Geographical and Natural Resources Research, Chinese Academy of Sciences
Soil properties	1∶1,000,000	200×200 m	Soil physical and chemical properties	Institute of Soil Science, Chinese Academy of Sciences
Weather	5 stations		Precipitation	China Meteorological Administration; Local bureau of Meteorology
Social economic data			Population, livestock rearing, fertilizer application	Field investigation; Statistics yearbook

### Model Description

The SWAT model [30] was selected to quantify the baseline NPS loads from each sub-watershed. To account for the spatial heterogeneity of climate, topography, land use and soil, we divided the study watershed into 47 sub-watersheds, with areas varying from 0.08 km^2^ to 4.61 km^2^ and an average area of 1.01 km^2^. A DEM was used to build a stream network (Fig. 1) and construct the spatial connections between sub-watersheds [31].

The hydrologic response units (HRUs) in SWAT are defined as the lumped areas by the land use, slope and soil type in a sub-watershed [32], [33]. As most of the equations are solved on the HRU level, 0% land use, slope and soil thresholds were chosen to define the HRU to capture small critical areas. A total of 141 HRUs were defined for the study watershed. To estimate the water balance and nutrient simulation, the curve number method and Modified Universal Soil Loss Equation were applied during the build-up period. Weather data (daily precipitation, minimum and maximum temperature, solar radiation and wind speed) were obtained from 14 state weather stations located approximately within the watershed. The pasture management information, such as the timing of manure and fertilizer application, grazing intensity and dates were collected from detailed interviews with local farmers. The sediment, N and P yields from each sub-watershed were subsequently routed through the channels to the watershed outlet, using the QUAL2E program.

The assessment point, at which the model parameters were evaluated [26], was placed at the Yulin station, which is situated at the watershed outlet (Fig. 1). Sensitivity analysis was performed to identify which parameters most influence outputs of interest. Based on the sensitivity analysis results, 28 parameters were modified using the Sequential Uncertainty Fitting version-2 program, which has been incorporated into SWATcup software [34]. In the Three Gorges Reservoir Region, the local government began periodic monitoring of N and P with approximately monthly sampling since 2004. Therefore, in this study, the monthly measured flow, sediment, N and P at the Yulin station for the period from 2004 to 2007 were used in the model evaluation. The calibration and validation was performed from January 2004 to December 2005 and January 2006 to December 2007, respectively. The Nash-Sutcliffe coefficient was used as a criterion to evaluate the model performance because it is the most common indicator in evaluating the hydrologic model [35].
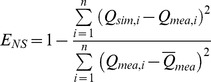
(1)Where, 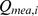 is the ith observation for the constituent being evaluated, 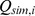 is the predicted value for the constituent being evaluated, 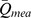 is the mean value of observed data for the constituent being evaluated, and n is the total number of observations.

### BMPs Scenarios

A calibrated SWAT program was operated for 5 years (2003–2007), and the simulation from 2003 was considered as a warm-up period to reduce the initial effects. The mean annual loads from 2004 to 2007 were qualified for all sub-watersheds as a baseline scenario. In scenarios for BMPs, the NPS loads were obtained from the baseline NPS loads and the removal percent from a BMP database [20], [36]. The calculation of NPS loads during the post-BMP period was numerically expressed using the following formula:
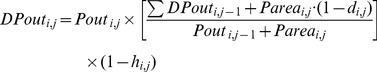
(2)Where *DP_out_* and *P_out_* is the NPS loads during the post- and pre-BMP periods, respectively, *P_area_* is the NPS load generated from the study sub-watershed during the pre-BMP period, *d* and *h* are the respective removal efficiencies for non-river BMP (wetland, detention pond and vegetative filter) and river BMP (ecological ditch), *j* and *j–1* represent the study and the upper sub-watersheds, respectively.

The structural BMPs considered in this study were detention ponds, wetlands, vegetative filter strips and grassed waterways. This selection was based on the history of BMPs implemented in the Yangtze watershed. To specifically address local characteristics, detention pond is designed as a permanent pool that is effective in retaining flow and trapping sediment and nutrient for certain time. Filter strip is designed as a uniformly-graded and densely-vegetated area which retards the surface runoff and controls the reel and sheet erosion. Wetland is supported to be an area covered partially or completely by shallow pools of water and grassed waterway is designed as a constructed watercourse consisting of vegetation to reduce runoff velocity, filter sediment, and absorb chemicals from sheet erosion. In conjunction with local characteristics, detention pond and wetland are designed to locate in the catchments and the capacity of detention ponds and wetlands were designed with 1- and 3-day hydraulic retention times, respectively. Vegetative filters and grassed waterways are planted at the riparian zone and along the watercourse of the corresponding stream in a sub-watershed. In conjunction with local characteristics, vegetative filters and grassed waterways were designed as 50% and 20%, respectively, of the length of the corresponding watercourse. The pollutant removal and cost data for each BMP were obtained from the BMP database (Table 2), which contains data collected from 275 BMPs [37]. The NPS loads and respective costs derived from the combination of BMPs were then dynamically integrated with the optimization engine at the watershed scale [38],[39].

**Table 2:** The information for selected BMPs based on a database containing 275 BMPs.

	BMP Type	Removal efficiency^1^ (%)			Cost information^2^
		Sediment	TP	TN	
1	Wetlands	71±25	56±35	19±29	C = 30.6V^0.71^
2	Detention pond	68±10	55±7	32±11	C = 24.5V^0.71^
3	Vegetative filter	38±31	14±23	14±41	$0.25–$0.50/ft^2^
4	Grassed watershed	54∼84	−25∼40	20	$0.30–$0.70/ft^2^

1 Data format: “mean ±95% confidence interval.”

2 V for the design capacity of structural BMP(ft^3^), C for the total cost(dollars).

### Topography Analysis

In this study, the sub-watershed was defined as a location variable constitutes a BMP or a set of BMPs. Therefore, the total number of variables equal to the sum of the combinations of the type, number and location of BMPs in each sub-watershed that needs to be optimally placed with BMPs. This hypothesis is reasonable because the sub-watershed is a suitable size for the installation of structural BMPs. The topographical features, in terms of the surface status, slope and the type of land use, were evaluated for each sub-watershed. The surface status indicator was defined as the degree of surface flatness, which could be quantified using the variance of elevation among the sub-watersheds [40]. The elevation data derived from a DEM were refined into uniform, homogeneous square grids (90 m*90 m), and the surface status indicator was calculated using the variance of elevation data obtained from each unit within a specific sub-watershed. The slope indicator was defined as the upland or channel slope, depending on the location of the structural BMP. The slope gradient was calculated by the elevations data and slope length of the drainage cells at the sub-watershed level [7]. The land use indicator was directly associated with the type of land use, which was determined from the land use - land cover map [41], [42]. In this study, the interpreted land use was classified into forests, paddy lands, grasslands, drylands, residential areas, bare lands and waters [29].

In the second step, these indicators were quantified using values 1∼10 in accordance with the local characteristics of each sub-watershed. When surface fluctuations are observed, additional land leveling and smoothing is needed; thus constructing BMPs in these areas is much more difficult. Therefore, a higher value was used when greater variance was observed (Table 3). The bare area was qualified as ‘1’ due to its convenience for engineers to implement a new structure project, whereas the residential land was defined as ‘9’ because it is more difficult to construct BMPs in this area (Table 4). Additionally, the structural BMPs will reach the expected efficiency at a range of optimal slope [37]. The literature-based optimal slopes obtained from the BMP database were <15° for wetlands, 2°∼6° for vegetative filters, <10° for detention ponds and 15° for grassed waterways (Table 5). The various topographical indicators were used as inputs for the optimization engine.

**Table 3:** The quantified result in accordance with the surface status.

Surface status (m)	value	Surface status (m)	value
0∼11	1	38∼47	6
11∼17	2	47∼57	7
17∼23	3	57∼70	8
23∼30	4	70∼92	9
30∼38	5	>92	10

**Table 4:** The quantified result in accordance with the land use pattern.

Landuse type	value	Landuse type	value
Bare land	1	Forest	5
Grass land	2	Paddy land	6
Waters	3	Towns, residential	7
Dry land	4		

**Table 5:** The quantified results in accordance with the degree of the slope.

Slope	Wetland	Detentionpond	Vegetativefilter	Grassed waterway
0°∼3°	5	5	2	2
3°∼6°	3	3	1	1
6°∼9°	2	2	2	1
9°∼12°	1	1	3	2
12°∼15°	1	1	5	3
15°∼20°	3	2	7	5
20°∼35°	5	3	9	7
35°∼60°	7	5	9	9
>60°	9	7	10	10

### Genetic Algorithm

The final objective function was designed as:
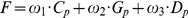
(3)Where *C_p_* is the total cost of the BMPs implemented in a watershed, *D_p_* is the annual NPS load at the watershed outlet, *G_p_* is the sum of the topography indicators at the watershed scale; *C_p_*, *D_p_* and *G_p_* are dimensionless values from 0 to 1 that were obtained after normalizing each objective; *w* are the weight values. In this study, five scenarios were designed corresponding to the different sets of weights. The five scenarios were as follows: 1) cost-effective-operative purpose; 2) cost-effective purpose; 3) sediment was the prior pollutant; 4) P was the prior pollutant; 5) N was the prior pollutant. The corresponding values of weights for each scenario could be seen in Table 6.

**Table 6:** The corresponding values of the *w* for each scenario.

Scenario	Description	*w* value					
		Cost	PollutantReduction	Operativity	Sediment	TP	TN
1	Multi-object	0.33	0.33	0.33	0.33	0.33	0.33
2	Cost-effect object	0.50	0.50	0	0.33	0.33	0.33
3	Sediment prior pollutant,	0.33	0.33	0.33	0.80	0.10	0.10
4	TP prior pollutant,	0.33	0.33	0.33	0.10	0.80	0.10
5	TN prior pollutant,	0.33	0.33	0.33	0.10	0.10	0.80

**Figure 2:**
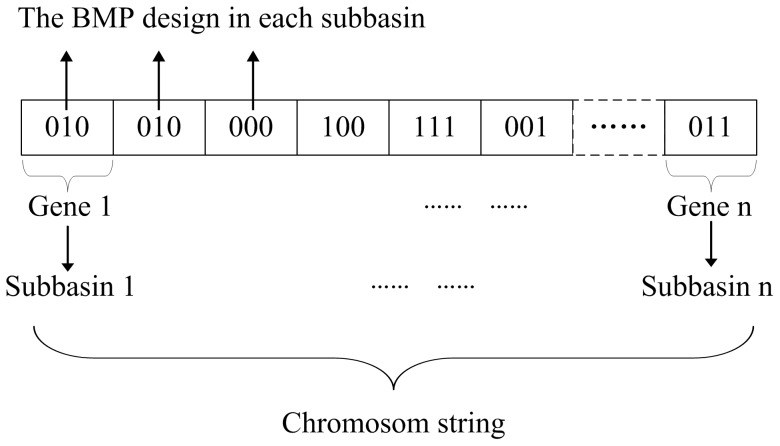
The population of chromosomes associated with the structural BMPs.

The genetic algorithm program, originally developed by Wall [43], was coded in Matlab to derive the final optimization. The sub-watershed was assumed as a variable coded in the form of genes [43], which represented either a single or a combination of BMPs (Table 7). The configuration of BMPs at the watershed scale was coded as the population of chromosomes (Fig. 2). The initial chromosomes were randomly generated for a given population size determined using a sensitivity analysis [20]. The individuals in the mating pool undergo crossover, which generates a population that exhibits the positive characteristics of the parents [44], and a mutation, which alters the chromosome state [45]. The solutions were selected or transferred into the next generation based on the following fitness equation:
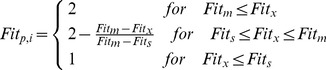
(4)Where *Fit_p,i_* (*C_p_*, *D_p_* and *G_p_*) is the value of the individual fitness, *Fit_m_* and *Fit_s_* is the maximum and minimum value of the parental population, and *Fit_x_* is the value of the present individual.

**Table 7:** The optimization design for the variables coded in the form of genes.

Gene Code	No	BMP design	Gene Code	No	BMP design
000	1	No measure	100	5	Ecological ditch
001	2	Wetlands	101	6	Vegetative filter and Wetlands
010	3	Detention pond	110	7	Vegetative filter and Detention pond
011	4	Vegetative filter	111	8	Vegetative filter and Ecological ditch

## Results

### The TAIOM Setup

**Figure 3:**
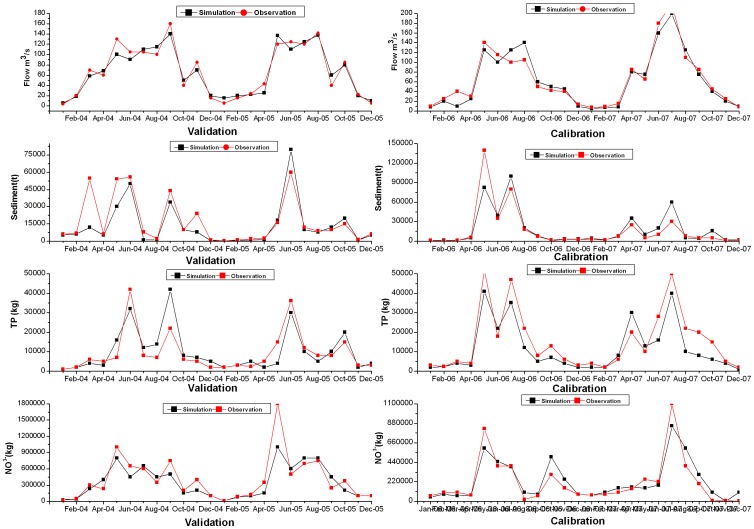
The goodness-of-fit results obtained during the calibration and verification period.

In the first step, we calibrated the SWAT model, quantified the topography indicator and genetic algorithm parameters to set up the TAIOM. As shown in Fig. 3, the observed time series flow closely matched with the simulated flow. The *E_NS_* value was 0.84 for the calibration period and 0.83 for the validation period. In the case of sediment and total N (TN) simulation, the monthly simulated value also matched well with the observed data in almost all seasons. The corresponding values of *E_NS_* were 0.81 and 0.83, respectively, for the sediment prediction, 0.80 and 0.77, respectively, for the TN simulation. However, the graphical plots of total P (TP) showed more differences between the simulated and measured data. The simulated peaks were lower than the observed peaks during the wet season, which might reflect the input or model errors [29]. The *E_NS_* values during the calibration and validation period were 0.70 and 0.69, respectively. To reduce the subjectivity in model evaluation, performance ratings were applied in accordance with Moriasi et al. [46]): very good (0.75–1), good (0.65–0.75), satisfactory (0.50–0.65), and unsatisfactory (≤0.5). Compared with other applications [29], [32], the sediment and TN predictions were very good, while the TP modeling was judged to be good in the Yulin watershed.

**Figure 4:**
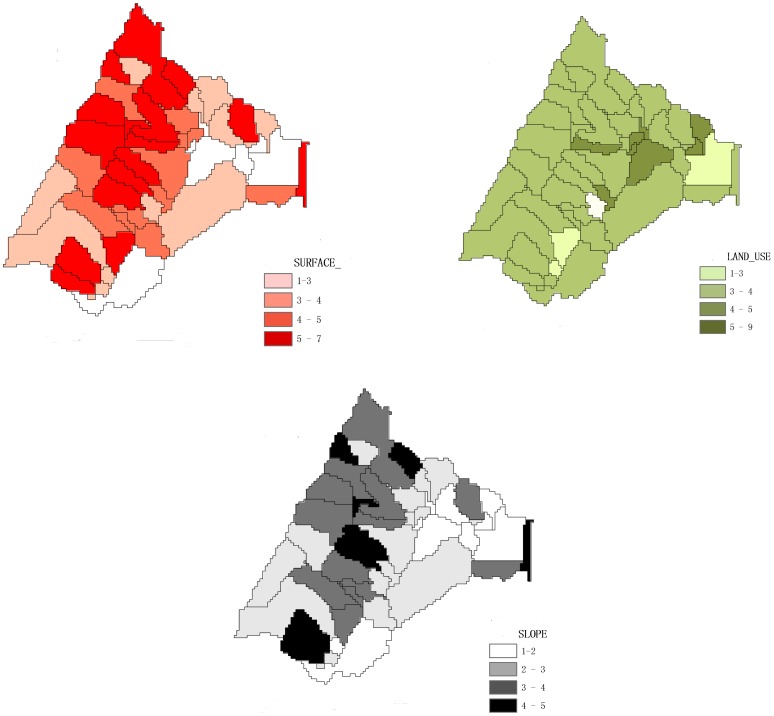
The spatial distribution of the quantified information for surface status, land use and slope.

The spatial distributions of the topography indicators are illustrated in Fig. 4. The higher values of surface and slope indicator were distributed in the western regions, containing mountainous areas and steep gorges. Lower values were concentrated in eastern regions, comprising relatively flat landscapes. With respect to the land use indicator, the values were uniformly distributed. The relatively high values distributed in the central part of the Yulin watershed indicated that there were more paddy lands and human activities in these areas.

**Figure 5:**
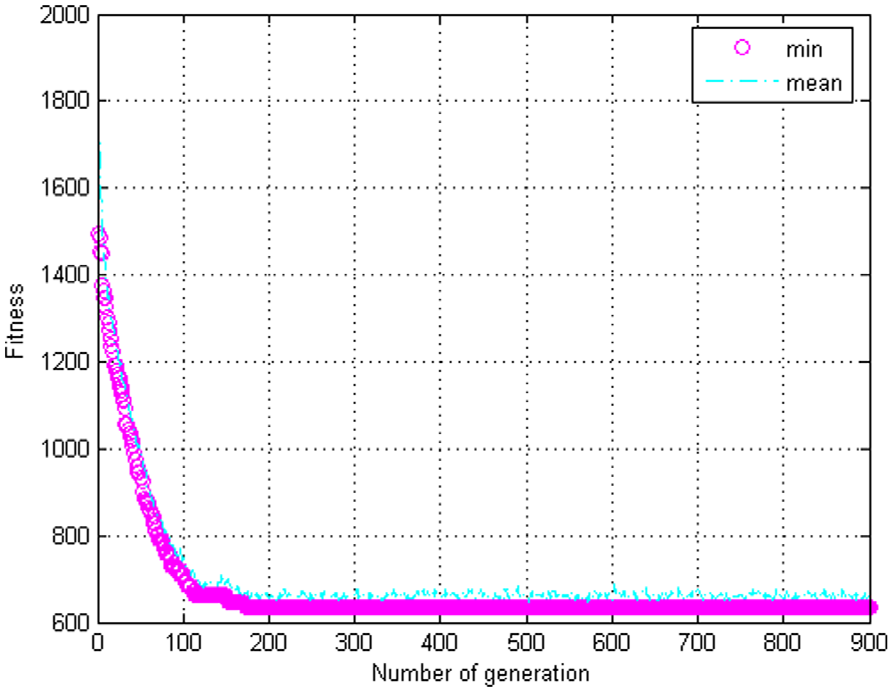
The sensitivity analysis related to genetic algorithm parameter.

To effectively determine the final solutions, the optimal genetic algorithm operational parameters were estimated. Initially, the genetic algorithm operational parameters were individually incremented using different population sizes and numbers of generations. As can be seen in Figure 5, the fitness dropped dramatically at 200 generations and only slight changes were observed when the genetic algorithm program was run for 1,000 generations. Additionally, the genetic algorithm performance increased significantly when the population was further increased to a population of 40, which reflects the increased freedom of the solution space [44], [45]. Finally, a total of 1,000 generations and a population of 40 were used in the final optimization. The entire process was completed in 30 minutes using a Centrino Duo processor running at 2.8 GHz.

### The TAIOM Results

**Figure 6:**
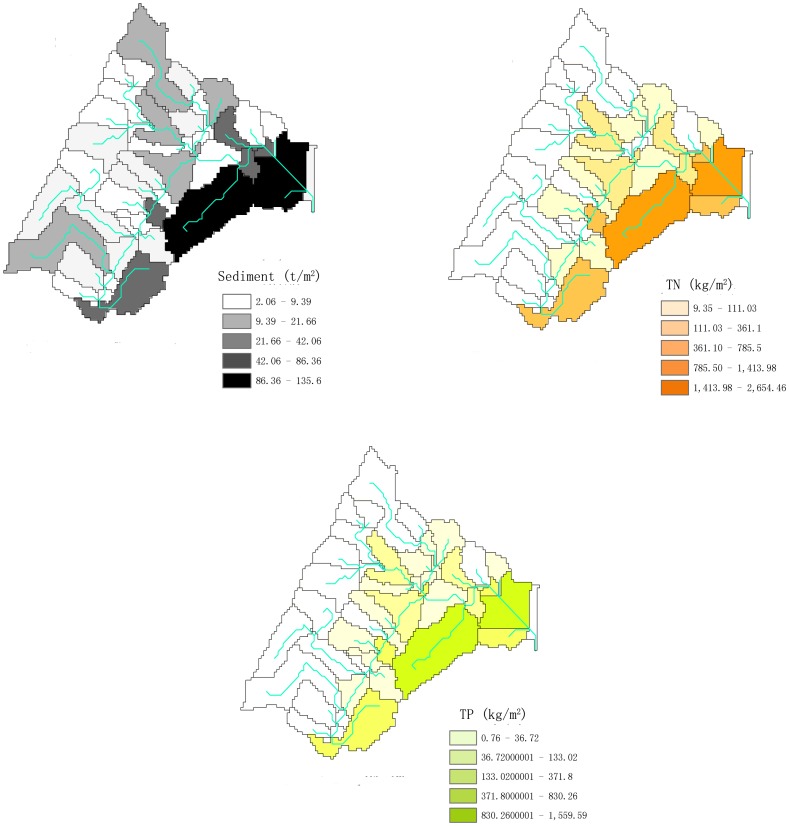
The sediment, NPS-TP and NPS-TN load during the pre-BMP period.

**Figure 7:**
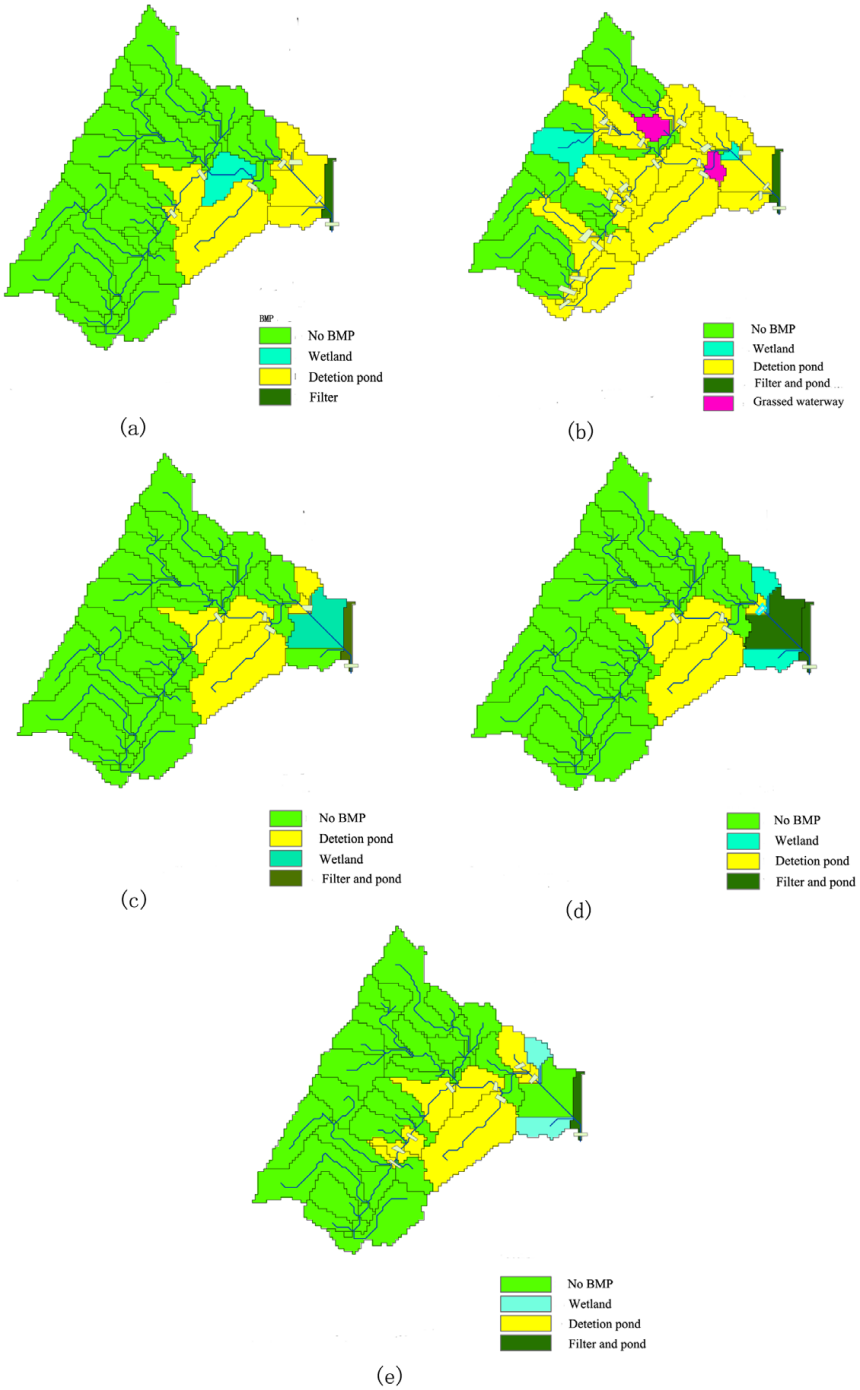
The spatial distribution of the structural BMPs for each optimization scenario.

The baseline simulated outputs and optimization results are shown in Table 8. The spatial distributions of the NPS loads during the pre-BMP period are further illustrated in Fig. 6, while the locations of the selected BMPs are compared in Fig. 7. In the baseline scenario, the sediment, TP and TN load at the watershed outlet were 2393.00, 3.22 and 97.18 ton yr^−1^, respectively. The sources of sediment were unevenly distributed in the Yulin watershed, with the highest load in the west, followed by the north and south regions, and the least in the east area. The sources of N and P were concentrated in the catchments of the middle- and down-stream.

**Table 8:** The TAIOM NPS (Sediment, NPS-N and NPS-P) load obtained during the post-BMP period.

Scenario	Sedimentt/a	TP t/a	TN t/a	Cost(10^6^ $)	Topographyindex
baseline	2393	3.22	97.18		
1	40.4	0.23	19.2	3.30	42.33
2	33.7	0.20	15.2	7.31	141.27
3	30.9	0.18	21.8	3.90	40.89
4	29.4	0.17	21.3	4.50	44.75
5	53.2	0.28	16.5	4.90	51.59

In scenario 1, the exported loads of sediment, TP and TN from the study watershed were reduced to 40.42 ton yr^−1^, 0.23 ton yr^−1^ and 19.23 ton yr^−1^, respectively, indicating respective reductions of 98%, 92% and 80%. The respective cost and sum of the topography indicators were $ 3.30×10^6^ and 42.33, respectively. The selected BMPs, in terms of 8 detention ponds, 1 wetland and 1 filter, were distributed throughout the landscapes near the watershed outlet (Fig. 7a). In scenario 2, the sediment, TP and TN loads at the watershed outlet were 33.70 ton yr^−1^, 0.20 ton yr^−1^ and 15.20 ton yr^−1^, respectively. Compared with those during the pre-BMP period, the respective removal percentages were 99%, 93% and 84%. The total cost and sum of the topography indicators were $ 7.30×10^6^ and 141.27, reflecting the combination of 25 ponds, 2 wetlands, 3 filters and a single grassed waterway (Fig. 7b).

As illustrated in Table 8 and Fig. 7c–7d, the effectiveness and spatial patterns of the selected BMPs in scenarios 3 and 4 were similar. In scenario 3, the exported sediment, TP and TN loads were 30.90 ton yr^−1^, 0.18 ton yr^−1^ and 21.80 ton yr^−1^, respectively, with respective removal percentages of 98%, 94% and 77%; in scenario 4, the load values were 29.40 ton yr^−1^, 0.17 ton yr^−1^, 21.30 ton yr^−1^, respectively, with respective removal percentages of 98%, 94%, 78%. In scenario 3, there were 7 detention ponds, 1 wetland and a single grassed waterway (Fig. 7c), whereas in scenario 4 there were 6 detention ponds, 3 wetlands and 2 grassed waterways (Fig. 7d). However, when N was chosen as the prior pollutant (scenario 5), the exported sediment, TP and TN loads were reduced to 53.24, 0.28 and 16.59 t yr^−1^, respectively, with respective reductions of 97%, 91% and 83%. In scenario 5, 11 detention ponds, 2 wetlands and a single grassed waterway were needed as illustrated in Fig. 7e.

## Discussion

### The Necessity of Topography Analysis

In this study, scenario 1 was designed to represent the TAIOM, while scenario 2 was designed to represent the traditional cost-effective method which had been studied for decades [5], [26], [28], [47]. Overall, the results of scenario 1 appeared to be more cost-effective than those of scenario 2 but greater reductions could be obtained in scenario 2 for all targeting pollutants. In this study, the application area for BMPs was defined as the sum of the sub-watershed area upon which the structural BMPs were installed [5], [48]. In scenario 2, the reduction amount per area was 0.83 t ha^−1^ for sediment, 1.10 kg ha^−1^ for TP, 29.00 kg ha^−1^ for TN, respectively. The reduction amount per unit cost were 0.32 t $^−1^ for sediment, 0.01 t $^−1^ for TP and 0.11 t $^−1^ for TN. In scenario 1, the reduction amounts per area were 2.34 t ha^−1^, 2.90 kg ha^−1^, 75.00 kg ha^−1^, respectively, with respective reductions per unit cost of 0.70 t $^−1^, 0.01 t $^−1^, 0.24 t $^−1^. This indicated that when the topography indicators were added, the optimization results were more cost-effective than the traditional method.

Similarly, all of the selected BMPs in scenario 1 were distributed throughout the landscapes near the watershed outlet, where relatively flat agricultural plains and gentle slope are primarily abundant (Fig. 7a). Therefore, the results of scenario 1 were considered to be reasonable because the key sources of N and P were concentrated in the same region (Fig. 6). In the Three Gorges Reservoir Region, high levels of human activities in these areas, such as the damage to the upper soil due to plowing and the high application of organic fertilizers, resulted in the increased leaching of N and P during the high-flow season [49]–[52]. Moreover, except for longer hydraulic retention times, these relatively flat areas also provide engineers with better construction conditions for the selected BMPs [29]. In scenario 2, the selected BMPs, were more evenly distributed in the Yulin watershed, with the most in the west, followed by the north and south regions, and the least in the east area (Fig. 7b). Further analysis demonstrated that the locations of BMPs in the east and south region were associated with the sources of sediment, which could be featured drylands and steeper slopes (Fig. 6). A comparison between Fig. 5 and Fig. 7a–7b showed that the slopes of these locations varied with a wide range of 0–60°, 3–35° and 3°–35° for detention ponds, wetlands and grassed waterways, respectively. Furthermore, 6 detention ponds and 1 wetland were distributed in the east region in scenario 2, which featured mountainous areas. Obvious surface fluctuations could be observed in these areas, and the engineers would need to perform additional land leveling and surface smoothing [40]. The results of scenario 1 were more operationally effective, with a mean topography indicator (per BMP) of 4.2, than the traditional method, with a value of 5 for a single BMP implementation.

### The Effect of Preference for Specific Pollutants

Scenarios 3–5 provide the descriptions of public preferences for specific pollutants. Table 8 indicates a combination of structural BMPs for reducing all pollutants to certain levels. Among those listed, wetlands and detention ponds were the most preferred BMPs in all cases. Many studies have described detention ponds and wetlands as permanent pools that are effective in reducing peak flow and trapping NPS pollutants [16], [53], [54]. As shown in Fig. 7, grassed waterways and filter strips were the least preferred in the Yulin watershed, as these areas are densely-vegetated areas at the watercourse or the border of the field [5], [55], [56]. In the Yulin watershed, the channel and catchment gradients were superior, indicating that the flow passes quickly and a portion of pollutants remain untreated, retarding flow velocity. The results of this study support the idea that increasing the water storage capacity through flow retention is an effective method for NPS control in the Three Gorges Reservoir Region.

As shown in Fig. 7, the controls for sediment and P were similar, reflecting the fact that most organic P attaches to sediment [57], [58]. Therefore, soil erosion is important for the production and transportation of P. However, as shown in Fig. 7d–7e, the controls for P and N were different. A comparison between scenarios 4 and 5 inferred that if the P strategy was implemented first, additional BMPs were required to achieve N removal in conjunction with the P scheme. Therefore, this study supports the idea that a single optimization result alone might not adequately reduce all the pollutants to the required levels [25], [28], [59].

### The Applicability of TAIOM

In this study, the GIS-based topography analysis can facilitate the rapid identification of potential locations for specific BMP installation. Rather, the surface status, slope and land use indicators were selected, which are essential to the Yulin watershed. However, the topography indicator might vary among watersheds [24]–[26]. The inclusion of more criteria depicting local topography characteristics for a specific watershed mediates the adaptation of the TAIOM to the study watershed. Certainly, except for topography indicators, the preferences for specific pollutants were also derived from detailed interactions with local stakeholders and engineers. Therefore, not all biases and subjectivity of the engineers have been removed in the process of the TAIOM. Within the scope of this paper, the TAIOM supports the selection process as a static state instead of subjective personal experience and judgment [24]–[26], [60].

Apart from the obvious advantage in terms of computational speed [20], [36], the use of the percentage removal calculation may be a limitation for TAIOM. Clearly, the sum of sub-watershed loads is not necessarily equal to pollutant yields at the outlet [20], [36]. In the particular case of the Yulin watershed, the in-stream process was neglected due to severe rainfall events and a large hydrographic gradient along the channel [61]. Therefore, the extent of the in-stream biogeochemical reaction was limited in the Yulin watershed. However, if the nutrient loads at the outlet do not match the total nutrient yields from the catchments in a different watershed, the calculations of downstream pollutant propagation should be included in the TAIOM framework.

### Conclusions

In this paper, a TAIOM was designed based on the integration of a topography analysis with the traditional cost-effective function to provide economically, ecologically and operationally effective solutions. Based on the results obtained from this study, the proposed TAIOM was more cost-effective than the traditional method. Within the TAIOM framework, all of the selected BMPs were distributed throughout landscapes that featured by relatively flat agricultural plains and gentle slopes, suggesting a more operationally effective scheme when the topography indicator was added. The TAIOM model developed in this study can be easily extended to other watersheds to develop the Total Maximum Daily Loads program. However, future works are required, which incorporate new criteria and more efficient optimization techniques.
